# Libel Suit

**Published:** 1856-04

**Authors:** 


*Libel Suit.*—In our last number we stated the result of a libel-suit instituted against this Journal by John D. Hill, and said that we might or might not, publish a report of the trial. We have been urged by very many friends to furnish such a report, and inasmuch as the subject is one in which our friends have a right to advise, we feel authorized to devote a few lines to an explanation of our reasons for withholding an account of the trial.

The first impulse was to publish a full account of the evidence and the speeches of the counsel, (for which we have all necessary material) in the belief that it would justify our position, and cast merited opprobrium on the other party. But subsequent reflection has convinced us that we have no special need of justification. We have no reason to believe that we have lost one friend by this suit; we know that we have gained many. Consequently, the only earthly importance this matter possesses with us is the money it costs us, together with the consideration that Prof. Flint is unfairly involved in the loss.

To go to the trouble of transcribing some fifty pages of manuscript, and the expense of printing it, would be only tolerable were some principle to be defended; but to expend all that labor in accomplishing a mere purpose of revenge upon an individual for whom our readers care nothing, and toward whom we have found it utterly impossible to get up any feeling approaching the dignity of hatred, would be anything but a labor of love. Here we drop the subject.

